# Non-Contact Video-Based Neonatal Respiratory Monitoring

**DOI:** 10.3390/children7100171

**Published:** 2020-10-06

**Authors:** Scott L. Rossol, Jeffrey K. Yang, Caroline Toney-Noland, Janine Bergin, Chandan Basavaraju, Pavan Kumar, Henry C. Lee

**Affiliations:** 1 Department of Pediatrics, School of Medicine, Stanford University, Stanford, CA 94305, USA; jkyang@stanford.edu (J.K.Y.); ctn@stanford.edu (C.T.-N.); jmbergin@stanford.edu (J.B.); hclee@stanford.edu (H.C.L.); 2 CocoonCam, Sunnyvale, CA 94089, USA; chandan@chryscloud.com (C.B.); pkpn@acm.org (P.K.)

**Keywords:** neonatal monitoring, respiratory rate, clinical alarms, video recording, biomedical technology

## Abstract

Respiratory rate (RR) has been shown to be a reliable predictor of cardio-pulmonary deterioration, but standard RR monitoring methods in the neonatal intensive care units (NICU) with contact leads have been related to iatrogenic complications. Video-based monitoring is a potential non-contact system that could improve patient care. This iterative design study developed a novel algorithm that produced RR from footage analyzed from stable NICU patients in open cribs with corrected gestational ages ranging from 33 to 40 weeks. The final algorithm used a proprietary technique of micromotion and stationarity detection (MSD) to model background noise to be able to amplify and record respiratory motions. We found significant correlation—*r* equals 0.948 (*p* value of 0.001)—between MSD and the current hospital standard, electrocardiogram impedance pneumography. Our video-based system showed a bias of negative 1.3 breaths and root mean square error of 6.36 breaths per minute compared to standard continuous monitoring. Further work is needed to evaluate the ability of video-based monitors to observe clinical changes in a larger population of patients over extended periods of time.

## 1. Introduction

The drive and control of breaths is an intricate system involving the circulatory, pulmonary, and central nervous systems. Chemoreceptors throughout the body monitor for hypoxia and hypercarbia, altering respiratory rate (RR) to maintain organ perfusion and optimal pH. Evidence has shown RR to be a better predictor of cardio-pulmonary deterioration than blood pressure and pulse rate [1]. In the neonatal population, ventilation and appropriate pulmonary support is critical. Effective ventilation is the key objective in neonatal resuscitation, and RR is included in all early warning systems for neonatal sepsis and necrotizing enterocolitis [2,3].

Traditionally, the gold standard for measuring RR is counting breaths for a minute while auscultating the patient or palpating for chest rise. While this is the most accurate, it is time consuming and not practical for an intensive care unit where continuous vital signs monitoring is required. Since the late 1800s, multiple innovations have been developed to automate measurements of respiration [4]. Early inventions measured breaths by airflow into a device, spirometry, or by attaching a circumferential strap around the chest, respiratory inductance plethysmography (RIP). Spirometry, however, is designed to measure lung volumes, and RIP is currently used in neonates to detect airway obstruction and for sleep apnea monitoring as it accurately records respiratory waveforms over time [5]. While RIP does measure RR, it requires bulky chest straps and interpretation from a pulmonologist or somnologist. 

Similar to RIP, impedance pneumography (IMP) measures changes in electrical signal secondary to the movements of the chest and diaphragm and has been widely adapted to multiple clinical settings. IMP uses mathematical algorithms to convert other electronic signals such as pulse oximeters and electrocardiograms (ECGs) to a respiratory wave form and RR, while RIP is derived directly from the attached straps. IMP can be integrated into standard continuous monitoring systems used by most hospitals [6]. While any manipulation of data or signal can introduce error, studies have shown strong correlation between RR gathered from RIP and IMP [6,7].

Nevertheless, there are several limitations of IMP. Information has to be gathered from electronic leads attached directly to the skin, and the signal needs to be amplified to be measured, making it susceptible to artifacts or noise. Artifacts and signal noise can originate from inadequate attachment of the electronic leads as well as any movement of the patient not related to breathing. In the neonatal population, these limitations are particularly apparent and produce several unique adverse outcomes. The frequency of false alarms secondary to the frequent movement of newborns is associated with provider alarm fatigue, infant hearing loss, and a disruptive environment for development [8,9,10,11,12]. Additionally, the humid environment of neonatal incubators and the infant’s thin, underdeveloped skin cause the adhesive in electric leads to fail and require frequent changing. The recurrent application of adhesive to the fragile premature skin causes breakdown and inflammation of their dermal barrier, introducing possible sources for infection [11].

Several innovations have been developed for non-contact monitoring in neonatology to minimize risk of dermal injury and alarm fatigue in infants. A variety of techniques have been studied from radio wave signaling, ultrasound, imaging photoplethysmography (PPG), and video-based respiratory monitors. In controlled environments, they have been shown to correlate with ECG monitoring with correlation coefficients from 0.79 to 0.92 [13,14,15,16]. Video-based respiratory monitors are particularly versatile due to the accessibility of cameras. These systems could be easily integrated into current monitoring systems and potentially used in clinics and at home for virtual medical appointments. However, due to the subtle motion of neonatal respiration, these technologies have struggled to accurately determine RR as the amplification of movement also increases signal noise, making it difficult to obtain an accurate measurement.

We conducted a course of study focused on a video-based respiratory monitoring system that extracts a respiratory waveform and rate without augmenting the infant’s surrounding or attire. Unlike past studies, this system extracts data on fully clothed or swaddled infants in a neonatal intensive care unit (NICU) with a variety of lighting and camera orientations. Through an iterative approach, we demonstrate a proprietary technique that is able to compensate for background signal noise while amplifying and measuring RR.

## 2. Materials and Methods

This study was performed at a single center, Lucile Packard Children’s Hospital at Stanford University. Patients in the neonatal intensive care unit (NICU) and step-down intermediate care nursery were enrolled in this study. Institutional Review Board (IRB) approval was obtained through the Stanford University Institutional Review Board. All monitoring was performed with written informed consent from parents and guardians in the NICU. Patients who were in an open crib, without significant complications, and not already enrolled in another study were eligible to enroll. Convenience sampling was used, with subjects recruited based on attending physician referral of eligible patients. Infants with concern for active infection, need for supplemental oxygen therapy, or vasopressors were excluded. To ensure patient safety, routine medical care and protective measures were not altered during monitoring sessions. Data being obtained from the study were not made available to clinicians during the course of their care for patients.

### 2.1. Study Design and Measurements

This manuscript describes an iterative design process. The objectives of the design were to identify and measure the respiratory rate of a preterm infant within an open crib through the analysis of camera footage and comparison to current hospital monitoring standards. The iterative process involved sequential algorithm design and application on recorded data. The principle analysis of the study investigated the consistent ability to identify patients within the frame and ability to extract the subjects’ respiratory rate. The secondary analysis compares respiratory rate from our non-contact monitoring to that recorded into the electronic medical record (EMR) by the current hospital standard of respiratory monitoring by ECG impedance pneumography. Any design that could not identify the patient, extract an RR for a majority of subjects, or did not correlate with the current standard was determined to be unsatisfactory, and the study would return to the previous design phase.

**Figure 1:**
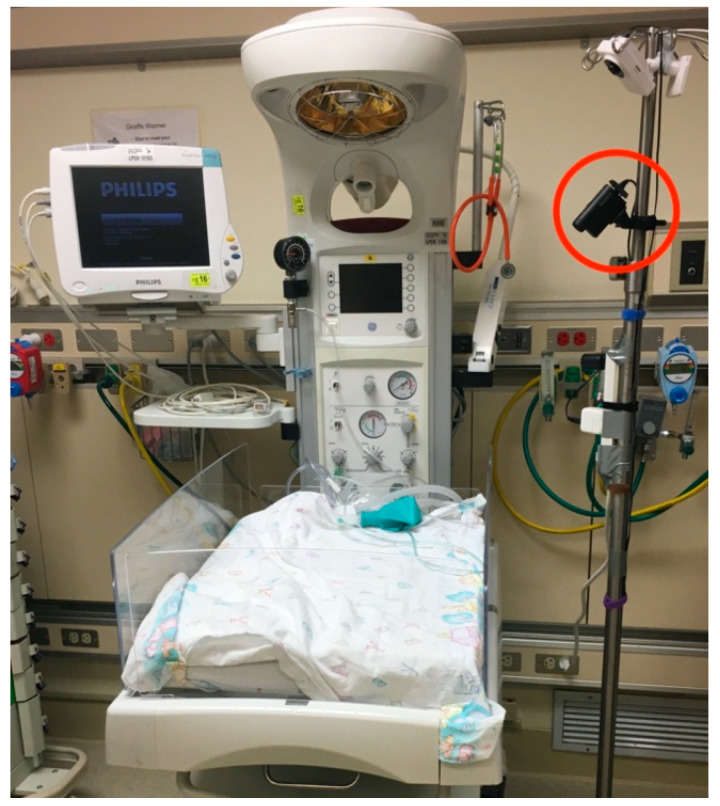
Study setup. Red circle shows camera set up above an open neonatal intensive care units (NICU) crib. The hospital-standard monitor can be seen on the opposite side of the crib as the camera.

Data were collected at the bedside as continuous 48-h video recordings from each infant. Footage was recorded on off-the-shelf IP cameras from Wansview with 320X180 resolution at ten frames per second and provided raw frames in YUV format. Cameras were placed approximately 4–6 feet from the bassinet (Figure 1). Simultaneously, vital sign data were collected for usual patient care from the ECG contact-based sensors and were extracted through the hospital’s Research Data Export system. No changes were made to the patient’s care while enrolled in this study.

### 2.2. Analysis

In the initial analysis, algorithms were assessed for consistent ability to identify the location of the patient in the crib, whether the patient was breathing, and finally if the monitoring could track respiration long enough to generate a respiratory rate. 

Analysis was based on the assumptions that the camera or crib did not move or sway and that the only moving element in the frame was the patient (i.e., no mobiles, moving toys or moving devices were in the frame of the recording).

In the secondary analysis, RR measured by the algorithm was compared to that extracted from standard monitoring data at the same moment in time. Agreement between the two modes of measurements was analyzed using a Bland–Altman plot. The extent and significance of correlation between the algorithm and standard monitoring were performed via linear regression. Finally, assuming complete accuracy of the ECG impedance pneumography measurement of respiratory rate, errors of algorithm measurements were determined by calculating the root mean square error.

## 3. Results

### 3.1. Subject Demographics

Eighteen subjects were enrolled and recorded in the study for the period of 2016–2017; one was excluded from final analysis due to conflicting documentation. At the time of recording, all infants were under the chronologic age of 10 weeks and had corrected gestational ages of 33–40 weeks. The mean age of patients at time of recording was 5 weeks and 35.5 weeks adjusted. The mean gestational age at birth was 30 weeks and 3 days. The population was 65% male and included a variety of racial backgrounds, with the largest group being White at 35%. Race was defined per electronic medical record with Asian including individuals of Indian and Southeast Asian descent. All subjects had various comorbidities of prematurity, with the prevalence of the most pertinent—apnea of prematurity, history of respiratory distress syndrome, chronic lung disease, and anemia of prematurity—demonstrated in Table 1. 

### 3.2. Interval Processing

The design process went through three cycles creating and testing three different algorithms: Eulerian video magnification followed by motion extraction, principal flow field, micromotion and stationarity detection. The final algorithm, micromotion and stationarity detection, achieved the design goals of consistently providing an RR for the subject and went on to the secondary analysis, comparing it to the continuous vital signs recorded into the subject’s EMR. 

#### 3.2.1. Eulerian Video Magnification Followed by Motion Extraction

The initial technique for extracting RR from footage was an Eulerian video magnification (EVM) algorithm to amplify movement, followed by a motion history image (MHI) algorithm to extract the motion.

EVM amplification has been described in other fields [17]. Our algorithm decomposes the video into pyramids of Laplacian images of different spatial frequencies to allow for greater accuracy and large amplification of minute motion. Laplacian pyramids are commonly used in motion magnification [18]. After pyramids were made, a low pass filter was performed and pixel intensity was increased based on each pyramid level. The amplified reconstructed frame was then superimposed on the original frame.

The MHI algorithm then generated the motion between frames by referencing successive binary silhouette images of the baby. The motion gradient between frames was used to calculate the amplitude for the inspiration/expiration signal of the baby’s breath and generate a continuous respiratory waveform. The respiratory rate could then be calculated from the waveform. The process of quantifying motion in a MHI is a unique process and has not been studied to our knowledge.

The benefits of the EVM and MHI approach was its ability to magnify small motions, with minimal processing and few artifacts. The critical deficits were the high rates of false positive signals. This was primarily from amplification of noise generated by changes in lighting and infant movement. 

#### 3.2.2. Principal Flow Field

Principal flow field (PFF) methodology was used in hopes of decreasing the impact of noise and increasing processing speed. Flow fields are a common way to evaluate movement in engineering. To optimize processing speed, our PFFs were performed on segments instead of full frames. This was accomplished by calculating optical flow fields by generating pixel movement gradients between frames. Knowing that inhalation and exhalation would be in the opposite direction allowed us to form flow field matrices localized to pixels representing respiration rather than noise.

PFFs were computed using the flow field matrices on the initial frame and adapted for each sequential frame. The PFF was used to generate a continuous respiration signal, which again was used to generate RR.

The benefit of PFF was its localized respiratory movements which decreased processing times while picking up similarly minute movements as the EVM algorithm. However, this algorithm was limited due to recurrent false positive readings with no infant present in the crib. This was due to the optical flow fields picking up the best plausible signal that represented respiratory movement even if this motion was actually noise from video capture, video compression, movement artifacts, or lighting changing. As there is always some level of noise, this algorithm would generate respiratory signals and rates even if a baby was not breathing or even if not present in the frame. 

#### 3.2.3. Micromotion and Stationarity Detection (MSD)

The final algorithm utilized micromotion and stationarity detection (MSD) and has not previously been studied for this application. To overcome the challenge of finding respiratory motion at a frame-to-frame level without incidentally measuring noise, the MSD analyzed and modeled the noise instead of trying to eliminate it.

In order to model noise characteristics, the image was divided into small sub-regions where changes in pixel intensity were measured over time. The model of the noise consisted of standard deviation (SD) measures of the changes in pixel intensity.

Assuming that the SD of the change in pixel intensity over a series of frames, with no motion and only noise, would remain relatively small and equal from frame to frame, then a large change in the SD of pixel intensity would indicate a micro movement. This is how imperceptible movements of chest rise and fall could be located and measured.

**Figure 2:**
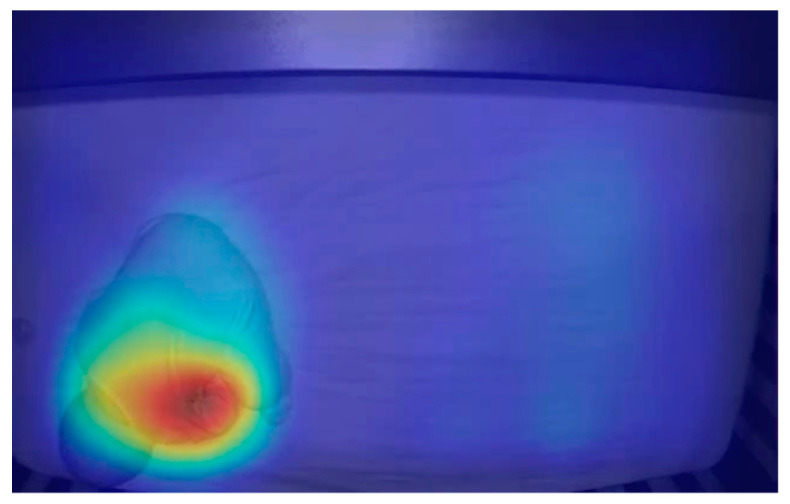
Heat map derived from standard deviation (SD) measures of SD measures showing motion due to breathing. The red region represents high SD measures and the blue region represents low SD measures. The red region was concentrated near the baby’s chest, indicating that the measurement showed motion associated with breathing.

Pixel intensities, or breathing motions, were calculated by taking SD measures of the SD measures. This generated heat maps that represented motion (Figure 2). To determine an RR, the number of peaks were counted and averaged over 100 frames of SD measured values.

A significant benefit of the MSD is its insusceptibility to noise and capability to detect whether or not an infant is in the frame and if that infant has had an apneic event, defined as a pause in breathing for greater than 20 s. The MSD algorithm had difficulty with measuring RR while the patient had gross or macro movements, such as movement of arms, leg, or torso with crying or shifting while sleeping. After such movements, the algorithm needs to recalibrate over 100 frames, or 10 s at 10 frames per second, to ensure it has located the subject and is measuring the correct signal. ECG impedance pneumography also cannot extract RR with large patient movements, but recovers more quickly after only a couple of seconds.

### 3.3. Respiratory Rate Correlation between ECG and MSD Video-Based Monitoring 

The secondary analysis was completed by running the MSD analysis on two patients. Their 48 h of video recording was scanned for continuous time frames where the patient remained asleep, relatively still, and unobstructed by staff or parents providing care. The combined continuous uninterrupted video consisted of 21 min and 50 s and contained 246 time points.

The MSD algorithm takes 10 s to calibrate, as described above, and then populates an RR every 5 s. The EMR produced a time point every 1 s as long as there was no interruption in the signal. To assure the two data sets represented the same points in time, the RR of 10 sequential timestamps from the video algorithm were assessed against the same time stamp from the EMR, and if there was a large discrepancy, then that series would be considered inaccurate and would not be included in the final data set. After confirmation that the time stamps matched up, respiratory rates from the MSD algorithm were compared to the RR from the EMR at the same corresponding time stamp until there was another interruption in either signal that prevented the two RRs from being generated at the same time. This assessment would then repeat to find the next usable segment of data.

**Figure 3:**
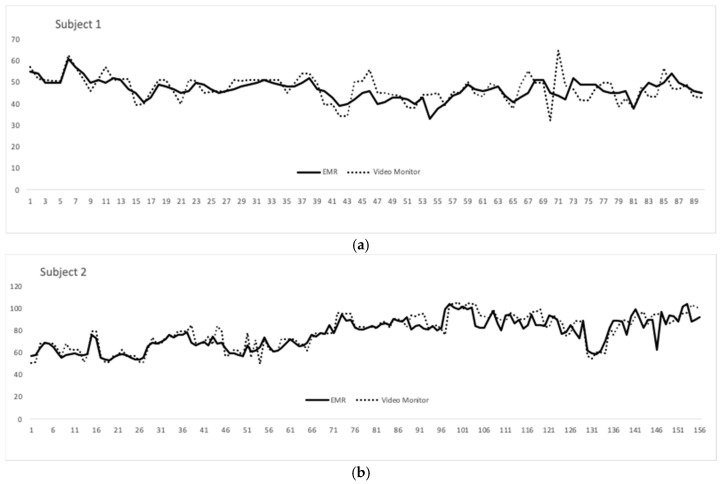
(**a**) Respiratory rate (RR) (y-axis) from the video monitoring system compared to that of the extracted Electronic medical record (EMR) data over a 8.4 min recording made up of 90 time points (x-axis); (**b**) RR (y-axis) from the video monitoring system compared to that of the extracted EMR data over a 13.4 min recording made up of 155 time points (x-axis)

The average RR over the approximately 22 min was 65 breaths per minute (BPM) in the EMR data and 67 in the video monitor group. The standard deviations were 18.4 and 19.7, respectively. Both patients recorded were male and Caucasian. Their mean gestation age at birth was 29 weeks and 5 days. Their mean adjusted age at recording was 36 weeks and 2 days. Overlying tracings between the EMR and video monitor over the recording time (in seconds) for both subjects are shown in Figure 3. 

**Figure 4:**
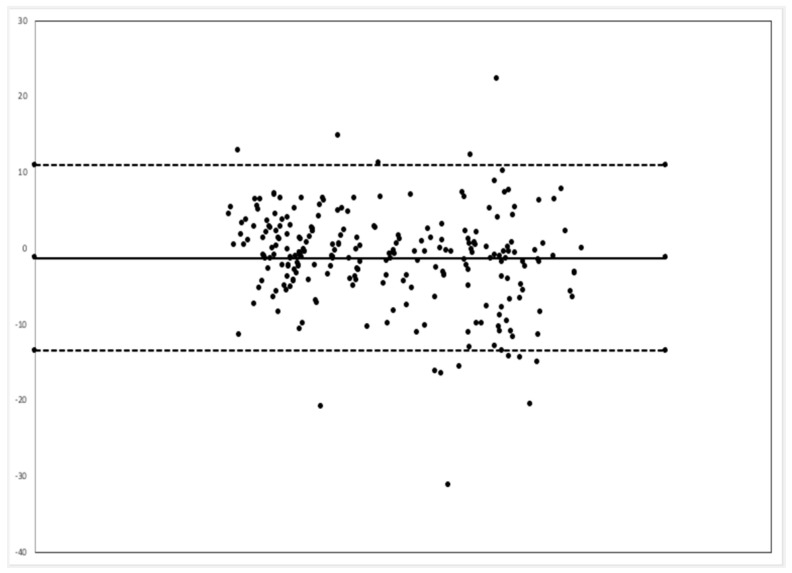
Bland–Altman plot. The central dark line represents a bias of −1.3 breaths per minute (BPM). The dashed lines represent the upper limit of agreement (10.9 BPM) and lower limit of agreement (−13.5 BPM).

Comparison between the EMR and MSD respiratory rate via the Bland–Alterman method showed that the video monitoring system had a bias of 1.3 less breaths per minute, and 94.3% of all time point comparisons were between the upper and lower limits of agreement (Figure 4).

**Figure 5:**
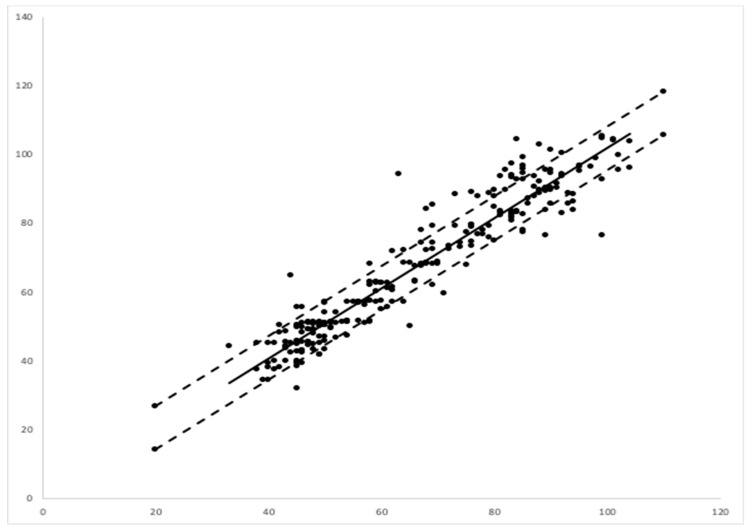
Linear regression comparing video-based monitoring respiration rate (RR) (y-axis) vs. electronic medical record (EMR)- RR (x-axis). The dashed lines represent upper and lower boundaries of root mean square error between the modes of measurement.

A linear regression between the EMR data and those of the camera-based non-contact monitor showed a correlation coefficient or multiple R of 0.948, with a *p* value of 0.001. The regression showed an R squared of 90%. Assuming that the EMR data were representative of the true respiration rate, the error of the video-based monitoring system was calculated as 6.36 breaths per minute via a root mean square analysis (Figure 5).

### 3.4. Limitations

The main limitation of this study is the relatively small sample size, short recording time, and lack of gold-standard comparison. While correlations are encouraging for the 246 time points recorded, the data were limited to a small population and short time period. As the patients in the secondary analysis were both Caucasian, the lack of diversity potentially limits the generalizability of the technology on a broader population level. This limitation may be mitigated by the technology’s capacity to detect RR through varied infant attire and different color swaddles. Lastly, by selecting relatively stable infants for 48 h of monitoring, our study was not designed to assess whether video monitoring systems can track clinically significant trends in respiratory rate. As this monitor was not available for the clinical team, it could not be assessed whether it had more or less false alarms or missed apneic or tachypnea events. 

## 4. Discussion

Video-based respiratory monitoring is at the earliest stages of development. However, our study shows the potential for MSD, making this technology equivalent to induction pneumography for at least a stable neonate in an open crib. While there are still a lot of qualifiers to its comparison, it is a major step toward a future without contact respiratory monitoring. Video-based monitoring could be integrated into incubator designs and attached to preexisting hospital cribs. With further validation, this technology could decrease the need for cardiac leads in more stable infants and be an adjunct monitoring tool for critically ill patients as a means to possibly decrease false alarms from patient movement.

A next major step for video-based respiratory monitoring systems is to validate the technology in a large cohort study inclusive of a diverse population with varying skin tone, race, sex, age, and clinical stability. Once validated, conducting studies that allow providers to view RR in real time would demonstrate the ability of video-monitoring to screen for apneas and tachypnea in infants. To be trusted in clinical settings, this technology must be shown to be as sensitive as induction pneumography. While preliminary data from this study suggest that it is as sensitive and specific as ECG-based systems, a longer and larger study focused on comparing significant events between the two systems is needed. There may also be opportunities for this technology to reduce false positive alarms, a known issue for hospitalized patients, either by being a more accurate monitor or by providing negative feedback to current monitors.

As MSD amplifies and tracks movement by averaging background noise, it has an advantage over induction pneumography which often falls short because of increased noise with faster respiratory rates. A future study comparing video-based respiratory monitoring and ECG induction pneumography to the gold standard of counting breaths for a minute in clinically ill and tachypneic patients could potentially show superiority of video-based systems in monitoring RR.

With the majority of pediatric illnesses being respiratory-related and the accessibility of high-quality cameras, such as those present in most personal cell phones, a video-based monitoring system could also have a large impact on outpatient medicine. It is conceivable that this technology could allow healthcare providers to measure RR throughout a telehealth video visit. This would provide valuable information that would aid in the decision-making process of whether or not a patient needs to go to an emergency room or would be safe to be cared for at home. With the SARS-CoV-2 pandemic increasing demand and showing the value of telemedicine visits, this technology could be invaluable.

## 5. Conclusions

Video-based monitoring using an MSD algorithm is a promising method to measure RR in the neonatal population. This preliminary study shows the arc of development and potential equivalency to the current industry standard of ECG induction pneumography. Further work is needed to continue to evaluate the ability of video-based monitors to record clinically relevant apneic events and other sudden physiological changes in comparison with current monitoring standards.

## Figures and Tables

**Table 1:** Demographics of enrolled subjects.

	Number	Proportion
**Sex**		
Male	11	0.65
Female	6	0.35
**Race**		
White	6	0.35
Hispanic/Latino	4	0.24
Asian	2	0.12
Native Hawaiian or Other Pacific Islander	2	0.12
Other	3	0.18
**Medical Issues**		
Apnea of Prematurity	13	0.76
History of Respiratory Distress Syndrome	11	0.65
Chronic Lung Disease	4	0.24
Anemia of Prematurity	15	0.88
	**Mean**	**SD ^1^**
Age at Recording (weeks)	5.3	3.1
Corrected GA ^2^ at Recording (weeks)	35.5	1.9
Birth GA ^2^ (weeks)	30.5	2.5
Height at Birth (cm)	40.4	3.8
Weight at Birth (kg)	1.4	0.4

^1^ Standard Deviation (SD); ^2^ Gestational Age (GA).
